# The associations of cerebrospinal fluid biomarkers with cognition, and rapid eye movement sleep behavior disorder in early Parkinson’s disease

**DOI:** 10.3389/fnins.2022.1049118

**Published:** 2022-11-23

**Authors:** Mingzhu Tao, Kaixin Dou, Yijie Xie, Binghui Hou, Anmu Xie

**Affiliations:** ^1^Department of Neurology, Affiliated Hospital of Qingdao University, Qingdao, China; ^2^Department of Clinical Laboratory, Affiliated Hospital of Qingdao University, Qingdao, China; ^3^Institute of Cerebrovascular Diseases, Affiliated Hospital of Qingdao University, Qingdao, China

**Keywords:** Parkinson’s disease, cognition function, rapid eye movement sleep behavior disorder (RBD), non-motor symptoms, cerebrospinal fluid biomarker

## Abstract

**Background:**

In Parkinson’s disease (PD), levels of cerebrospinal fluid (CSF) biomarkers and progression of non-motor symptoms are associated, but the specifics are not yet clear.

**Objective:**

The aim of this study was to investigate the associations of non-motor symptoms with CSF biomarkers in PD.

**Materials and methods:**

We assessed 487 individuals from the Parkinson’s Progression Markers Initiative (PPMI), consisting of 155 healthy controls (HCs) and 332 individuals with PD. Patients with PD were grouped according to non-motor symptoms and compared CSF α-synuclein (α-syn), amyloid-beta 1-42 (Aβ_1–42_), and total tau (t-tau) levels. Multiple linear regressions were used in baseline analysis and linear mixed-effects models in longitudinal analysis. Analyses of mediating effects between cognition and CSF biomarkers were also performed.

**Results:**

At baseline, PD patients with cognitive impairment (PDCI) exhibited significantly lower CSF α-syn (β = −0.1244; *P* = 0.0469), Aβ (β = −0.1302; *P* = 0.0447), and t-tau (β = −0.1260; *P* = 0.0131) levels than PD patients without cognitive impairment (PDCU). Moreover, a faster decline of α-syn (β = −0.2152; *P* = 0.0374) and Aβ (β = −0.3114; *P* = 0.0023) and a faster rise of t-tau (β = −0.1534; *P* = 0.0274) have been found in longitudinal analysis. The Aβ positive group showed an earlier decline in cognitive performance (β = −0.5341; *P* = 0.0180) compared with the negative Aβ group in both analyses. In addition, we found that PD patients with probable rapid eye movement sleep behavior disorder (pRBD) showed decreased CSF α-syn (β = −0.1343; *P* = 0.0033) levels. Finally, mediation analysis demonstrated that olfactory function partially mediated the relationship between cognition and CSF biomarkers levels.

**Conclusion:**

Our study shows that CSF biomarkers are associated with cognition at baseline and longitudinally. Cognitive impairment is more severe in patients with a heavier Aβ burden. CSF α-syn decreased in PD patients with pRBD. This study suggests that early recognition of the increased risk of non-motor symptoms is important for disease surveillance and may be associated with the pathological progression of CSF markers.

## Introduction

Parkinson’s disease (PD) is a chronic neurodegenerative disorder that affects the central nervous system, second only to Alzheimer’s disease (AD). The main pathological feature of PD is the deposition of α-synuclein (α-syn) and fibrillar translocation from the brainstem to the limbic system as formation of Lewy bodies (LBs) (Mattila et al., 2000; Irwin et al., 2013). Additional pathological changes such as amyloid-beta plaque and neurofibrillary tangles also act as factors in the progression of the disease process (Popescu et al., 2004; Nakashima-Yasuda et al., 2007; Clinton et al., 2010; Raunio et al., 2019). PD is clinically manifested with a broad spectrum of motor symptoms such as rigidity, bradykinesia, and resting tremor and non-motor symptoms such as cognitive impairment, sleep behavior disorder, and olfactory dysfunction. These non-motor symptoms generally precede the appearance of motor symptoms and are a risk factor for increased mortality (Levy et al., 2002; Aarsland et al., 2003; Hobson and Meara, 2004), and individual progression varies considerably over time (Hughes et al., 2000; Williams-Gray et al., 2007; Aarsland and Kurz, 2010).

Mild cognitive impairment (MCI) is defined as cognitive decline that can be observed through neuropsychological scales, in the intermediate stages of subjective cognitive decline (SCD) and dementia (Aarsland et al., 2021). Patients with PD have an almost six-fold risk bound to foster dementia than normal (Aarsland et al., 2001). Dementia is one of the most important factors for patients with advanced disabilities, which greatly reduces the quality of life. MCI in PD manifests itself more specifically as deficits in attention, executive function, and visuospatial processing (Emre et al., 2007). There are indications that deficits in different cognitive domains may have different abilities to predict dementia (Aarsland et al., 2021). Patients with PD and AD showed a high degree of pathological congruence, with a marked accumulation of β-amyloid plaques and total tau (t-tau) protein containing neurofibrillary tangles (Heywood et al., 2015). Recent studies had suggested that several biomarkers including α-syn, amyloid-β, and t-tau have been associated with cognitive impairment in PD (Bibl et al., 2006; Huang et al., 2006; Morley et al., 2012; Parnetti et al., 2014).

Rapid eye movement sleep behavior disorder (RBD) occurs during rapid eye movement (REM) sleep and manifests as enacting dreams and loss of muscle atonia, leading to violent, aggressive movements, and may injure oneself and a bed partner (Yamada and Ueda, 2019). The occurrence of RBD has a strong association with neurodegenerative diseases, especially with α-syn including PD, and dementia with Lewy bodies (DLB). Sleep behavior disorders were meaningfully connected with diminished CSF α-syn (Goldman et al., 2018; Mollenhauer et al., 2019; Wang et al., 2022) but most focus on RBD in PD cohorts. In this study, CSF α-syn is reduced in PD and distinguishes PD with pRBD from PD without RBD.

Biofluid markers can be useful in shedding light on the pathology and variability of PD in the long term. However, the deep correlation of non-motor symptoms with CSF biomarker levels has not been appropriately explored. Therefore, we modeled using data from the PPMI database to explore the association of non-motor symptoms with CSF biomarker levels in patients through baseline and longitudinal analysis. In addition, we investigated the possible links by using mediating effect analyses.

## Materials and methods

### Participants and characteristics

All data for this research were obtained from the PPMI database.^1^ The PPMI is a longitudinal, observational cohort study of participants with PD and healthy controls (HCs). It is beneficial to dig into further evidence supporting the diagnosis of PD and CSF biomarkers of progression and their intrinsic associations. Subjects underwent assessments including motor, non-motor, imaging, and donated biological samples including CSFs. Of the 495 enrolled subjects, 487 subjects (155 HCs and 332 PD) who agreed to donate CSF samples at baseline visits were included in our study. Subjects who were recruited into the PD group were at the disease threshold. They needed to meet the criteria of (1) untreated for PD and (2) having an asymmetric resting tremor or asymmetric bradykinesia or two of bradykinesia, resting tremor, and rigidity with a diagnosis within 2 years. For more details on the PPMI inclusion and exclusion criteria, please visit the official website. Every subject selected for the baseline analyses has completed the non-motor assessments [the REM Sleep Behavior Disorder Screening Questionnaire (RBDSQ), the Montreal Cognitive Assessment (MoCA), and the University of Pennsylvania Smell Identification Test (UPSIT)]. Moreover, to ensure the accuracy of the biomarker data, individuals with hemoglobin above the measurable value were excluded. To avoid fluctuations in the effect on olfaction and sleep, people with severe neurological disorders that may interfere with sleep and those who were taking antidepressants were excluded. Participants without baseline or follow-up data of CSF biomarkers (α-syn, Aβ_1–42_, and t-tau) were excluded. Participants were informed and written informed consent was obtained prior to the start of the study. All studies related to PPMI have been approved by the local Institutional Review Board prior to commencement.

The cognitive assessment included the MoCA, the Letter-Number Sequencing (LNS) Score, the Symbol Digit Modalities Test (SDMT) Score, and the Hopkins Verbal Learning Test Delayed Recall (HVLT-R). In this study, a MoCA score of 26 was used as a cutoff value to distinguish MCI groups, and the PD with cognitive impairment group was identified as a score less than the cutoff value (Litvan et al., 2012). This classification method takes the MCI in PD (PD-MCI) standard published in 2012. Based on the results of a recent assessment of the Aβ_1–42_ test for comparative analysis, we took 683.45 pg/ml as the threshold to distinguish between Aβ_1–42_ high and low groups (Shaw et al., 2019).

The RBDSQ questionnaire was used to screen for RBD (Mollenhauer et al., 2019). Using an overall RBDSQ score of 6 as the optimal threshold, a score of 6 or higher could be recognized as pRBD (Nomura et al., 2011).

### Biomarker measurements

The collection and processing of cerebrospinal fluid (CSF) follows a standardized procedure, refer to the PPMI Biologics Manual for details. Each subject’s CSF specimen corresponds to its unique code. The samples were frozen and stored in the PPMI Biorepository Core Laboratory until they were transferred to the University of Pennsylvania Center for Bioanalysis at the time of analysis. We assessed CSF for α-syn (analyzed using an ELISA assay available commercially from Covance), Aβ_1–42_, and t-tau (measured using the electrochemiluminescence immunoassays on a fully automated cobas e601 analyzer).

### Data analysis

Statistical analysis and figures were performed using the R (version 4.1.0) software. Biomarker values with a right-skewed distribution underwent logarithmic α-syn, Aβ_1–42_, and t-tau transformations *via* the “car” package of R software. The mean ± standard deviation (SD) was used to represent continuous variables. The categorical variables were expressed as the number of patients and their percentage. Differences between groups were compared using the Mann–Whitney–Wilcoxon test for continuous variables and the chi-square test for categorical variables. The association between CSF biomarkers (t-tau, Aβ_1–42_, and α-syn) and cognition was analyzed at baseline using multiple linear regression equations. The model was corrected for age, gender, years of education, and hemoglobin when used. Further longitudinal analysis of cognition and CSF biomarkers were explored using linear mixed-effects models. The above two analyses are also applied in the analysis of the relationship between sleep behavior and α-syn. Multiple linear regression was used to calculate the correlation between the mean rate of change in CSF levels and the longitudinal change in cognitive correlation scale scores. The rate of change is obtained using the “arm” package in R software through 10,000 repetitions. To further explore the relationship of CSF biomarkers and cognition decline or α-syn and sleep behavior mediated by PD pathology, mediation analyses were used. The mediation effect is based on the method of causal-steps approach and the Sobel test. Furthermore, the indirect effect is estimated, with the significance determined using 10,000 bootstrapped iterations (“mediate” package in R 4.1.0 software). The indirect effect *P*-value was statistically significant.

The Bonferroni correction was used for multiple comparisons. All tests used a significance level of *P* < 0.05.

## Results

### Participant characteristics at baseline

Table 1 shows the baseline demographic data. In general, 332 PD (mean age 61.8 ± 10.0 years, 35.5% female) and 155 control subjects (mean age 61.2 ± 11.1 years, 36.8% female) were included in the analyses. No statistical difference was found between the control group and the total PD group in age (*P* = 0.98), gender (*P* = 0.79), and education year (*P* = 0.09). All participants were well-educated, with an average of approximately 15 years of education. Patients with PD exhibited a greater loss of olfactory function (UPSIT score; *P* < 0.01) and greater burden of cognition (MoCA score; *P* < 0.01) and sleep behavior disorder (RBDSQ total score; *P* < 0.01) compared with HCs. There were significant differences in processing speed (SDMT; *P* < 0.01) and delayed verbal recall (HVLT-R; *P* < 0.01) in cognitively related markers, except for working memory (LNS; *P* = 0.13). Control subjects had higher CSF Aβ_1–42_ (*P* < 0.01), α-syn (*P* < 0.01), and t-tau (*P* < 0.01) than those of the patients with PD.

**TABLE 1:** Baseline characteristics of included participants.

	Overall (*n* = 487)	Control (*n* = 155)	Total PD (*n* = 332)	*P*
Age, Mean (SD), years	61.7 (10.0)	61.2 (11.1)	61.8 (10.0)	0.9815
Female, N (%)	175 (35.9)	57 (36.8)	118 (35.5)	0.7923
Education, Mean (SD), years	15.7 (3.0)	16.0 (2.8)	15.6 (3.0)	0.0936
Disease Duration, Mean (SD), years	NA	NA	6.7 (6.5)	NA
MDS-UPRDS III, Mean (SD)	NA	NA	20.7 (8.9)	NA
MoCA, Mean (SD)	27.5 (2.0)	28.2 (1.0)	27.2 (2.3)	**<0.0001**
LNS, Mean (SD)	10.6 (2.6)	10.9 (2.6)	10.5 (2.7)	**0.1341**
SDMT, Mean (SD)	42.7 (10.0)	46.3 (10.8)	41.1 (9.1)	**<0.0001**
HVLT-R, Mean (SD)	8.7 (2.5)	9.3 (2.3)	8.3 (2.5)	**<0.0001**
RBDSQ, Mean (SD)	3.7 (2.6)	2.8 (2.3)	4.1 (2.7)	**<0.0001**
UPSIT, Mean (SD)	26.1 (9.1)	34.0 (4.7)	22.4 (8.3)	**< 0.0001**
CSF Aβ_1–42_, Mean (SD), pg/ml	972.8 (415.5)	1068.0 (494.6)	928.3 (365.9)	**0.0079**
CSF α-syn, Mean (SD), pg/ml	1601.6 (662.0)	1719.6 (696.9)	1545.7 (639.7)	**0.0044**
CSF t-tau, Mean (SD), pg/ml	179.8 (61.7)	195.3 (74.9)	172.6 (53.1)	**0.0033**

PD, Parkinson’s disease; control healthy control; *P* < 0.05 for total PD vs. controls; SD, standard deviation; MDS-UPDRS III, Movement Disorder Society-sponsored Unified Parkinson’s Disease Rating Scale part III; MoCA, Montreal Cognitive Assessment; LNS, Letter Number Sequencing Score; SDMT, Symbol Digit Modalities Score; HVLT-R, Hopkins Verbal Learning Test Delayed Recall; RBDSQ, Rapid-eye-movement sleep Behavior Disorder Screening Questionnaire; UPSIT, University of Pennsylvania Smell Identification Test; CSF, cerebrospinal fluid; Aβ_1–42_, amyloid-β_1–42_; α-syn, α-synuclein; t-tau, total tau; NA, not available. Bold values represent the the *P*-value < 0.05.

### The relationships of cognition level with cerebrospinal fluid biomarker in cross-sectional analyses

**FIGURE 1:**
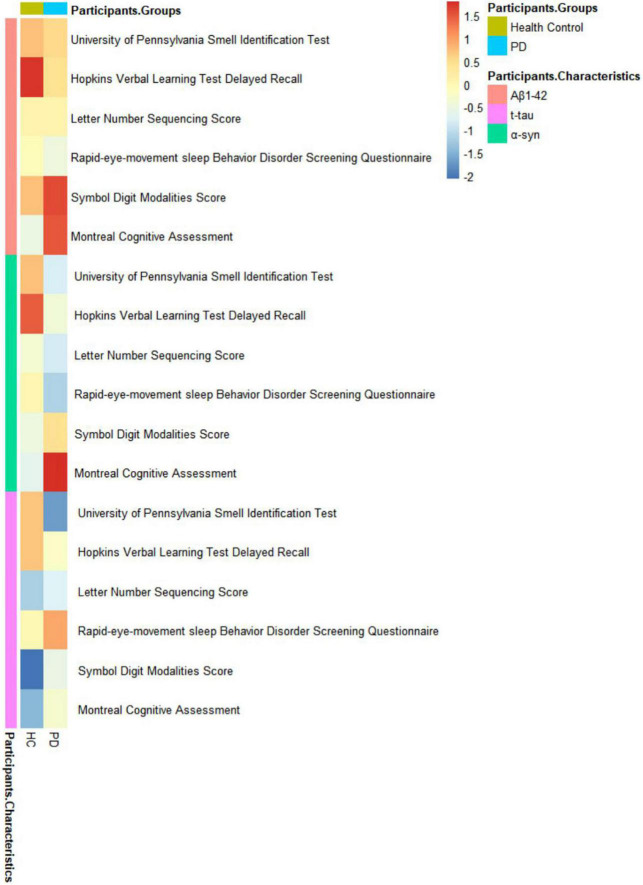
Associations of non-motor symptoms with cerebrospinal fluid (CSF) biomarker levels in cross-sectional analyses.

**FIGURE 2:**
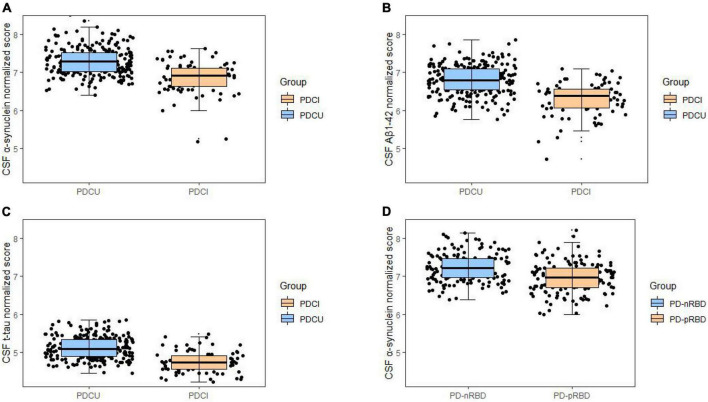
Associations of cognitive impairments with lower cerebrospinal fluid (CSF) biomarker levels in cross-sectional analyses. Cognitive impairments were significantly associated with lower levels in **(A)** α-synuclein **(B)**, Aβ_1–42_, and **(C)** t-tau. Cross-sectional associations of sleep disorder with lower CSF **(D)** α-synuclein were shown. *P < 0.05.

The relationships between CSF biomarkers and cognitive impairment were revealed (Figure 1). PD with cognitive impairment correlated lower levels of CSF biomarkers in α-syn (β = −0.1244; *P* = 0.0469) (Figure 2A), Aβ_1–42_ (β = −0.1302; *P* = 0.0447) (Figure 2B), and t-tau (β = −0.1260; *P* = 0.0131) (Figure 2C). These results were observed when we excluded subjects with high CSF hemoglobin (Hb) levels (>200 mg/ml) to avoid the potential impact of contaminant plasma Hb. In addition, PD individuals showed significant correlations between MoCA and CSF biomarkers. PD with cognitive impairment who had higher MoCA scores was significantly associated with higher CSF α-syn (β = 0.0494; *P* = 0.0107) (Supplementary Table 4) and Aβ_1–42_ (β = 0.0310; *P* = 0.0089) (Supplementary Table 4). Besides, a significant association between MoCA scores and lower CSF α-syn levels was found in PD without cognitive impairment (β = −0.0244; *P* = 0.0330) (Supplementary Table 4). Conversely, there was no significant association between the cognitive-related scale and CSF biomarkers in individuals with PD.

Furthermore, in the comparison of measured cognition performance, PD patients with low CSF Aβ_1–42_ showed significantly lower MoCA (β = −0.5556; *P* = 0.0411) (Supplementary Table 2) scores and higher SDMT (β = 2.2793; *P* = 0.0384) (Supplementary Table 2), whereas working memory (LNS) and delayed verbal recall (HVLT-R) did not exhibit significant differences.

### The effects of cognition decline at baseline on cerebrospinal fluid biomarkers during the follow-up

**FIGURE 3:**
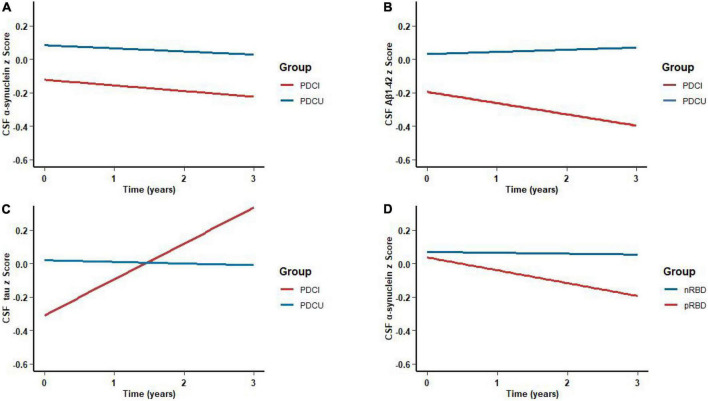
Associations of cognitive impairments with lower cerebrospinal fluid (CSF) biomarker levels during the follow-up. Temporal trajectories of CSF **(A)** α-synuclein **(B)** and Aβ_1–42_ show a decline in cognitive impairments. However, CSF **(C)** t-tau was elevated. Longitudinal associations of sleep disorders with CSF **(D)** α-synuclein were shown. *P < 0.05.

A total of 332 (PD patients without cognitive impairment, PDCU: *n* = 284; PD patients with cognitive impairment, PDCI: *n* = 48; high CSF Aβ_1–42_: *n* = 244; low CSF Aβ_1–42_: *n* = 88) individuals were included in the model fit during 3-year follow-up beyond baseline measurements, including at least one follow-up measurement. In a linear mixed model (LME) adjusted for age, gender, education, and baseline diagnosis, the cognition impaired predicted a decline in CSF biomarker level over time. Compared with PD patients without cognition impairment group, the PD patients with cognition impairment were significantly associated with Aβ_1–42_ (β = −0.3114; *P* = 0.0023) (Figure 3B), α-syn (β = −0.2152; *P* = 0.0374) (Figure 3A), and t-tau (β = −0.1534; *P* = 0.0274) (Figure 3C). Associations between cognition level and CSF biomarkers in PDCU participants and PDCI participants are shown in Supplementary Table 5. Results revealed significant associations between MoCA and t-tau (β = 0.0086; *P* = 0.0362) in PDCU; α-syn and MoCA (β = −0.0090; *P* = 0.0037) in PDCI; and SDMT and Aβ_1–42_ (β = 0.0042; *P* = 0.0391) in PDCI, but not in all participants with PD.

The low CSF Aβ_1–42_ patient group showed a more pronounced downward trend in the test of global cognitive functioning (MoCA) (β = −0.5341; *P* = 0.0180) (Supplementary Table 3) and working memory (LNS) (β = −0.8590; *P* = 0.0341) (Supplementary Table 3) than those with high CSF Aβ_1–42_. However, we found no significant difference in processing speed (SDMT) (β = 0.9204; *P* = 0.1284) (Supplementary Table 3) and delayed verbal recall (HVLT-R) (β = 0.0812; *P* = 0.6637) (Supplementary Table 3).

### The relationships between sleep behavior disorder and α-syn

As for participants with PD, the probable rapid eye movement sleep behavior disorder (pRBD) group had lower CSF α-syn (β = −0.1174; *P* = 0.0412) (Figure 2D) biomarker levels than the non-RBD group. In the comparison of measured biomarker levels for 3 years, PD patients with high RBDSQ total scores showed significantly lower CSF α-syn (β = −0.1343; *P* = 0.0033) (Figure 3D) level, whereas Aβ_1–42_ and t-tau did not exhibit a significant difference.

### Mediation analyses

**FIGURE 4:**
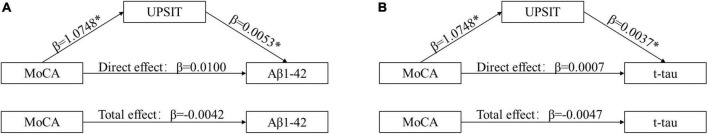
Olfactory dysfunctions mediate the effects of Montreal Cognitive Assessment (MoCA) on cerebrospinal fluid (CSF) biomarkers. Causal-step approach showed that (1) MoCA was significantly associated with olfaction and rates of CSF biomarkers change; (2) olfaction was associated with CSF biomarkers; (3) when controlling for olfaction, the associations between MoCA and rates of CSF biomarkers were still significant (*P* < 0.05). As such, all conditions for mediation were met. For Aβ_1–42_ **(A)** and t-tau **(B)**, the Sobel test for mediation estimated that the percentages of total effect mediated by olfaction were 35% (*z* = 2.1965; *P* = 0.024) and 37% (*z* = 0.7674; *P* = 0.026), respectively. *P < 0.05. MoCA, Montreal Cognitive Assessment; UPSIT, University of Pennsylvania Smell Identification Test; CSF, cerebrospinal fluid; Aβ_1–42_, amyloid-β_1–42_; t-tau, total tau.

The above results indicated that there are associations between cognitive decline and CSF biomarker levels. Olfactory impairment is the earliest non-motor symptom to appear in PD and is not easily detectable. Therefore, mediated analysis was used to explore the mechanisms involved in cognitive decline with olfactory symptoms. We demonstrated that the MoCA scores were significantly associated with the UPSIT scores (β = 1.0747; *P* = 0.0001) and that the UPSIT scores were significantly associated with CSF Aβ_1–42_ (β = 0.0054; *P* = 0.0163) (Figure 4A). Moreover, when controlling for olfaction, the association between cognition decline and CSF biomarkers was no longer significant. Similarly, the UPSIT scores were significantly associated with CSF t-tau (β = 0.0037; *P* = 0.0340) (Figure 4B), and cognition and CSF t-tau were also not significant when we controlled olfactory function. This suggested that olfactory function acted as a partial mediator in the relationship between cognition and CSF biomarkers. However, there were no significant associations between RBD and α-syn when using the UPSIT scores as a mediator to analyze.

## Discussion

Based on the results of the analysis, an association was shown between biomarker levels and progression of non-motor symptoms. At this stage, CSF biomarkers cannot be used alone as diagnostic and typing criteria for the diagnosis of PD. However, assessing its association with non-motor symptoms provides a clue to the progression of non-motor symptoms and may explain the pathological progression of the CSF marker.

Our analyses demonstrated that cognition has a high predictive value for progressive reductions in CSF Aβ_1–42_ and α-syn levels. In contrast, CSF t-tau levels increased with disease progression. All three CSF biomarkers were lower in PDCI than in PDCU at baseline. At the same time, baseline CSF Aβ_1–42_ level can also predict cognition function changes. PD patients with low CSF Aβ_1–42_ showed a faster decline of MoCA and SDMT than those with high CSF Aβ_1–42_ at baseline. Only the MoCA and LNS are meaningful in the CSF grouping in longitudinal data analysis. Besides, PD with RBD shows low CSF α-syn levels.

Consistent with our findings, a decrease in CSF α-syn was observed in patients with PD, and further results were found in the study that analyzed a complete dataset of patients with cognitive impairment who showed greater changes over the course of the disease than those without cognitive impairment (Baek et al., 2021). On the contrary, an increase in CSF t-tau was observed in patients with PD with a duration of more than 5 years without dementia (Hall et al., 2016). This variance can be due to one or more factors of several. Various characteristics of the case population or the different analysis methods used to measure the CSF biomarkers were included. The intraneuronal Lewy inclusions formed with α-syn as the main component are the main pathological feature of PD. A series of changes in α-syn misfold, aggregate, and deposit occur earlier than motor symptoms (Jellinger, 2014; Berg et al., 2021). During the onset of PD, it has been found that t-tau protein interacts with α-syn, intra-axonal α-syn, and intraneuronal t-tau aggregates associated with axon disease and cellular dysfunction (Li et al., 2016). Specifically, α-syn oligomers induce the formation of toxic t-tau protein oligomers, and α-syn and t-tau proteins can synergize with each other to form fibrous amyloid structures (Sengupta et al., 2015). One study containing 129 postmortem human brain samples showed that synaptic proteins are associated with cognition (Bereczki et al., 2016). In our models obtained from the PPMI database, a consistent trend of change appears in CSF α-synuclein which declines overall in PD and is more pronounced in cognitive impairment and CSF t-tau, which rises overall in PD and is the same in cognitive impairment.

In fact, a considerable number of studies have shown that reduced Aβ_1–42_ level is the most common CSF biomarker associated with cognitive impairment (Siderowf et al., 2010; Compta et al., 2013; Alves et al., 2014; Parnetti et al., 2014; Lin and Wu, 2015). Diffuse amyloid-β plaques are higher in the striatum of PD with dementia (PDD) than in PD without dementia, and this striatal pathology has been suggested as a potential mechanism for cognitive decline (Kalaitzakis et al., 2008). The same decrease in amyloid-β levels may also be seen in patients with classic DLB. The midbrain substantia nigra has the largest group of dopamine neurons that provide a feedback loop to the striatum important for cognition levels (Braak et al., 2003; Kalia and Lang, 2015). Cortical dopamine modulation in healthy subjects can boost working memory as well as processing speed suggesting a core role in cognition function (Ranganath and Jacob, 2016; Westbrook et al., 2020). Recently, a previous study shows a decreased CSF Aβ_1–42_ level in PDD compared with PD without dementia or age-matched control subjects (Mollenhauer et al., 2006). A prospective cohort study with at least one yearly longitudinal follow-up evaluation supported that reduced CSF Aβ_1–42_ was an independent predictor of cognitive decline in patients with PD consistent with previous research (Siderowf et al., 2010), which also confirmed our results. Furthermore, a study analyzing neurodegenerative disease showed that Aβ_1–42_ had significant reductions in patients with early PD without dementia, and the reductions were associated with memory impairment but not with executive attention or visuospatial dysfunctions (Alves et al., 2010). Similar to our results, PD patients with low CSF Aβ_1–42_ levels showed worse cognitive performance. However, unlike in previous studies, there are no significant differences in delayed verbal recall and processing speed (Baek et al., 2021). The difference from previous analyses may be due to the inclusion of data and the analysis using equations. This study used a scale of cognitive correlation for patient changes in longitudinal lengths rather than cross-sectional data to analyze correlation changes. Our study shows a predictive value of CSF Aβ_1–42_ for global cognition.

Parkinson’s disease usually begins at the lower brain stem (dorsal motor nuclei) and olfactory bulb, according to Braak staging (Braak et al., 2004). A possible explanation for the α-syn pathology is that it initially affects the REM sleep-regulatory circuits in the caudal brain stem. There is still a lot of confusion over the mechanisms leading to the α-syn change in PD. Nevertheless, there seems to be a dynamic interplay between the aggregation of synaptic proteins as LBs and synaptic degeneration to explain the α-syn shifts in PD (Parnetti et al., 2019). Based on our results, PD patients with pRBD individuals had lower α-syn and a faster downward trend in the longitudinal analysis compared to those without RBD individuals. A previous study shows a correlation between a greater decrease in CSF α-syn levels and an increase in the incidence of RBD, in tandem with the mechanism of progression of synucleinopathy in pathology (Wang et al., 2022). This was consistent with our findings.

In addition, we observed a mediation effect of the UPSIT score on the relationship between global cognition and CSF biomarkers, although the exact mechanism was not clear. It has been reported that the progression of Aβ pathology in the olfactory bulb reflects the severity of respective pathologies in other brain areas, which results from large mortem studies (Attems et al., 2014). Olfactory dysfunction that occurs early in the course of the disease may be a marker for cognitive decline (Bohnen and Müller, 2013). An immunohistochemical study showed a clear association between olfactory alterations and tau protein pathology (Passali et al., 2015). A reasonable hypothesis would be global cognition that may indicate more Aβ pathology.

There are several limitations to our study. First, due to the PPMI database only including data from patients with early onset PD, our study lacked biomarker data for patients with advanced PD, which could have an impact on the accuracy of the longitudinal analysis. Changes in biomarkers may be masked. Second, the questionnaires assessing non-motor symptoms, including MoCA and RBDSQ, were self-reported without objective measures, so the results need to be further validated in the pathology. Third, cognition in patients with PD may worsen over time, becoming dementia or returning to normal, which also needs to be considered. In addition, there may be overlaps in the distribution of CSF biomarkers between PD groups with and without cognitive impairment, affecting their utility. Taken together, although the variability of this study was limited, our analysis showed that changes in CSF biomarkers were associated with cognitive decline in patients with PD, and pRBD would be associated with lower CSF α-syn. The PPMI study is expected to mature with long-term longitudinal follow-up observations that will resolve these limitations. Future research is needed to draw more robust conclusions and obtain new evidence on the underlying relationship between CSF biomarker levels and non-motor symptoms.

## Conclusion

Taken together, our study helps to reveal the association between non-motor symptoms and CSF biomarkers, specifically in cognitive impairment and Aβ_1–42_ as well as RBD and α-syn. In addition, olfaction may mediate the associations between CSF biomarkers and cognition. Changes in biomarkers may be associated with non-motor symptoms. These findings still need to be further analyzed and validated in future models that incorporate more sample-fit optimizations.

## Data availability statement

The data were sourced from the Parkinson’s Progression Markers Initiative (PPMI) database. The original contributions presented in this study are included in the article/Supplementary material, further inquiries can be directed to the corresponding authors.

## Author contributions

AX and BH responsible for the conception and design of the study. MT, KD, and YX performed the data analysis. MT and KD contributed to the design of the study and drafted the manuscript. All authors contributed to the article and approved the submitted version.

## Footnotes

1
http://www.ppmi-info.org
