# Supplementary material for: The associations of cerebrospinal fluid biomarkers with cognition, and rapid eye movement sleep behavior disorder in early Parkinson’s disease
Source: Front Neurosci. 2022 Nov 23;16:1049118. doi: 10.3389/fnins.2022.1049118 (PMC9728099; doi:10.3389/fnins.2022.1049118)
Supplement: Supplementary file 1 [file Data_Sheet_1.docx]

**Title: Associations of Sleep Disorders with Cerebrospinal Fluid α-Synuclein in Prodromal and Early Parkinson’s disease**

**CONTENT**

| **TITLE** |  | **PAGE** |
| --- | --- | --- |
| **Table S1** | Baseline characteristics of Parkinson disease | 2-3 |
| **Table S2** | Comparison of cognitive level in High CSF Aβ1-42 and Low CSF Aβ1-42 groups in cross-sectional study | 4 |
| **Table S3** | Comparison of cognitive level in High CSF Aβ1-42 and Low CSF Aβ1-42 groups in longitudinal study | 5 |
| **Table S4** | Associations of Non-motor symptoms with CSF biomarker levels in PD and control individuals in cross-sectional study | 6 |
| **Table S5** | Associations between rates of change in CSF biomarker and rates of change in cognition function | 7 |

**Table S1 Baseline characteristics of Parkinson disease**

|  | **High CSF Aβ1-42**  **(n=244)** | **Low CSF Aβ1-42**  **(n=88)** | **p** | **PDCU**  **(n=284)** | **PDCI**  **(n=48)** | **p** | **PD-pRBD**  **(n=125)** | **PD-nRBD**  **(n=207)** | **p** |
| --- | --- | --- | --- | --- | --- | --- | --- | --- | --- |
| Age, Mean (SD), years | 61.4(9.4) | 63.1(9.6) | 0.0927 | 61.9(9.7) | 61.8(8.6) | 0.6697 | 61.9(9.2) | 61.8(9.6) | 0.9432 |
| Female, N (%) | 89(36.5) | 29(33.0) | 0.5553 | 106(37.3) | 12(25) | 0.1056 | 37( | 81( | 0.0794 |
| Education, Mean (SD), years | 15.6(2.9) | 15.4(3.2) | 0.7285 | 15.7(2.9) | 15.0(3.7) | 0.2361 | 15.6(2.8) | 15.6(3.1) | 0.9053 |
| Disease Duration, Mean (SD), years | 6.4(6.3) | 7.3(7.0) | 0.2611 | 6.5(6.4) | 7.7(7.2) | 0.4810 | 6.3(6.3) | 6.9(6.7) | 0.2721 |
| MDS-UPRDS III, Mean (SD) | 20.6(8.6) | 20.9(9.6) | 0.9517 | 20.1(8.6) | 24.3(9.6) | **0.0024** | 20.2(8.6) | 21.0(9.0) | 0.5517 |
| MoCA, Mean (SD) | 27.0(2.3) | 27.5(2.1) | 0.1994 | 27.4(2.2) | 25.7(2.4) | **<0.0001** | 27.1(2.2) | 27.2(2.3) | 0.5688 |
| LNS, Mean (SD) | 10.5(2.7) | 10.6(2.5) | 0.6480 | 10.8(2.5) | 8.8(2.8) | **<0.0001** | 10.3(2.5) | 10.6(2.6) | 0.3256 |
| SDMT, Mean (SD) | 41.5(9.1) | 39.6(9.5) | 0.0781 | 42.1(8.6) | 34.9(10.0) | **<0.0001** | 39.8(8.8) | 41.7(9.2) | 0.1195 |
| HVLT-R, Mean (SD) | 8.4(2.5) | 8.2(2.7) | 0.8683 | 8.7(2.3) | 6.1(2.7) | **<0.0001** | 8.0(2.5) | 8.6(2.5) | **0.0401** |
| RBDSQ, Mean (SD) | 4.0(2.6) | 4.3(3.0) | 0.5421 | 3.9(2.6) | 5.0(3.1) | **0.0234** | 6.9(1.9) | 2.3(1.1) | **<0.0001** |
| UPSIT, Mean (SD) | 22.6(8.5) | 21.8(7.7) | 0.3460 | 22.6(8.2) | 21.3(8.8) | 0.3785 | 21.2(8.5) | 23.1(8.0) | 0.0561 |
| CSF Aβ1-42, Mean (SD), pg/ml | 1068.9(322.0) | 538.6(103.4) | **<0.0001** | 942.3(376.5) | 846.0(285.2) | 0.2033 | 946.7(390.1) | 917.3(349.1) | 0.4529 |
| CSF α-syn, Mean (SD), pg/ml | 1642.7(654.1) | 1276.9(511.7) | **<0.0001** | 1575.0(633.7) | 1372.5(440.0) | 0.1000 | 1537.2(651.2) | 1550.9(631.0) | 0.8762 |
| CSF t-tau, Mean (SD), pg/ml | 178.4(49.0) | 156.4(60.5) | **<0.0001** | 174.9(53.9) | 158.7(46.6) | **0.0722** | 177.9(53.4) | 169.4(52.6) | **0.0860** |

PD Parkinson’s disease, P < 0.05 for low CSF Aβ1-42 vs. high CSF Aβ1-42, PD with probable REM sleep behavior disorder vs. PD without REM sleep behavior disorder, PDCU vs. PDCI. PDCU cognitively unimpaired PD; PDCI cognitively impaired PD patients, SD standard deviation, MDS-UPDRS III Movement Disorder Society sponsored Unified Parkinson’s Disease Rating Scale part III, MoCA total scores of Montreal Cognitive Assessment, LNS Letter Number Sequencing Score; SDMT Symbol Digit Modalities Score; HVLT-R Hopkins Verbal Learning Test Delayed Recall, RBDSQ Rapid-eye-movement sleep Behavior Disorder Screening Questionnaire, UPSIT University of Pennsylvania Smell Identification Test, CSF cerebrospinal fluid, Aβ1-42 amyloid-β1-42, α-syn α-synuclein; t-tau total tau

**Table S2 Comparison of cognitive level in High CSF Aβ1-42 and Low CSF Aβ1-42 groups in cross-sectional study**

|  | **CSF Aβ1-42 level** | | |
| --- | --- | --- | --- |
|  |  |  |  |
|  | **β** |  | **p** |
| MoCA | -0.5556 |  | **0.0411** |
| LNS | -0.4516 |  | 0.1451 |
| SDMT | 2.2793 |  | **0.0384** |
| HVLT-R | 0.1046 |  | 0.7370 |

MoCA total scores of Montreal Cognitive Assessment; LNS Letter Number Sequencing Score; SDMT Symbol Digit Modalities Score; HVLT-R Hopkins Verbal Learning Test Delayed Recall.

†: adjusted for age, sex, years of education, and CSF hemoglobin; Bold text: significant results

**Table S3** **Comparison of cognitive level in High CSF Aβ1-42 and Low CSF Aβ1-42 groups in longitudinal study**

|  | **CSF Aβ1-42 level** | | |
| --- | --- | --- | --- |
|  |  |  |  |
|  | **β** |  | **p** |
| MoCA | -0.5341 |  | **0.0180** |
| LNS | -0.8590 |  | **0.0341** |
| SDMT | 0.9204 |  | 0.1284 |
| HVLT-R | 0.0812 |  | 0.6637 |

MoCA total scores of Montreal Cognitive Assessment; LNS Letter Number Sequencing Score; SDMT Symbol Digit Modalities Score; HVLT-R Hopkins Verbal Learning Test Delayed Recall.

†: adjusted for age, sex, years of education, and CSF hemoglobin; Bold text: significant results

| **Table S4 Associations of Non-motor symptoms with CSF biomarker levels in PD and control individuals in cross-sectional study** | | | | | | | | | |
| --- | --- | --- | --- | --- | --- | --- | --- | --- | --- |
|  | | **PD** | |  | **PDCU** | |  | **PDCI** | |
|  |  | **β** | **P^†^** |  | **β** | **P^†^** |  | **β** | **P^†^** |
| α-syn | MoCA | -0.0146 | 0.1090 |  | -0.0244 | **0.0330** |  | 0.0494 | **0.0107** |
|  | LNS | -0.0072 | 0.3520 |  | -0.0079 | 0.4040 |  | -0.0263 | 0.1900 |
|  | SDMT | 0.0046 | 0.0670 |  | -0.0030 | 0.2850 |  | 0.0079 | 0.1040 |
|  | HVLT-R | 0.0016 | 0.8521 |  | -0.0086 | 0.4260 |  | -0.0213 | 0.2470 |
| Aβ1-42 | MoCA | -0.0109 | 0.2660 |  | -0.0166 | 0.1585 |  | 0.0310 | **0.0089** |
|  | LNS | -0.0011 | 0.8900 |  | -0.0102 | 0.3260 |  | -0.00073 | 0.6670 |
|  | SDMT | 0.0049 | 0.0702 |  | 0.0021 | 0.5420 |  | 0.0063 | 0.2080 |
|  | HVLT-R | 0.0056 | 0.5400 |  | -0.0041 | 0.7320 |  | -0.0178 | 0.3410 |
| t-tau | MoCA | 0.0011 | 0.8746 |  | -0.0031 | 0.7257 |  | 0.0008 | 0.9073 |
|  | LNS | 0.0037 | 0.5491 |  | 0.0021 | 0.7825 |  | -0.0039 | 0.7570 |
|  | SDMT | 0.0027 | 0.1480 |  | 0.0009 | 0.7089 |  | 0.0014 | 0.6340 |
|  | HVLT-R | 0.0085 | 0.1980 |  | 0.0052 | 0.5628 |  | -0.0037 | 0.7460 |

PD: Parkinson’s disease; PDCU cognitively unimpaired PD; PDCI cognitively impaired PD patients; CSF: cerebrospinal fluid; α-syn α-synuclein; Aβ1-42 amyloid-β1-42; tau total tau; MoCA total scores of Montreal Cognitive Assessment; LNS Letter Number Sequencing Score; SDMT Symbol Digit Modalities Score; HVLT-R Hopkins Verbal Learning Test Delayed Recall.

†: adjusted for age, sex, years of education, and CSF hemoglobin; Bold text: significant results

| **Table S5 Associations between rates of change in CSF biomarker and rates of change in cognition function** | | | | | | | | | |
| --- | --- | --- | --- | --- | --- | --- | --- | --- | --- |
|  | | **PD** | |  | **PDCU** | |  | **PDCI** | |
|  |  | **β** | **P^†^** |  | **β** | **P^†^** |  | **β** | **P^†^** |
| α-syn | MoCA | -0.0021 | 0.5501 |  | 0.0026 | 0.5838 |  | -0.0090 | **0.0037** |
|  | LNS | 0.0013 | 0.7300 |  | 0.0043 | 0.3390 |  | -0.0115 | 0.1239 |
|  | SDMT | 0.0004 | 0.7252 |  | -0.0006 | 0.6406 |  | 0.0024 | 0.2091 |
|  | HVLT-R | 0.0012 | 0.7228 |  | 0.0054 | 0.2607 |  | -0.0073 | 0.1996 |
| Aβ1-42 | MoCA | -0.0001 | 0.9717 |  | -0.0025 | 0.5928 |  | 0.0019 | 0.7501 |
|  | LNS | 0.0023 | 0.5446 |  | 0.0001 | 0.9700 |  | 0.0074 | 0.3452 |
|  | SDMT | 0.0015 | 0.1916 |  | 0.0001 | 0.8957 |  | 0.0042 | **0.0391** |
|  | HVLT-R | -0.0006 | 0.8527 |  | -0.0043 | 0.3726 |  | 0.0023 | 0.7058 |
| t-tau | MoCA | 0.0003 | 0.8864 |  | 0.0086 | **0.0362** |  | -0.0028 | 0.4992 |
|  | LNS | 0.0015 | 0.5624 |  | 0.0054 | 0.1480 |  | -0.0019 | 0.7260 |
|  | SDMT | 0.0003 | 0.7084 |  | 0.0001 | 0.9379 |  | 0.0003 | 0.7957 |
|  | HVLT-R | -0.0011 | 0.6413 |  | -0.0024 | 0.4468 |  | -0.0007 | 0.8659 |

PD: Parkinson’s disease; PDCU cognitively unimpaired PD; PDCI cognitively impaired PD patients; CSF: cerebrospinal fluid; α-syn α-synuclein; Aβ1-42 amyloid-β1-42; tau total tau; MoCA total scores of Montreal Cognitive Assessment; LNS Letter Number Sequencing Score; SDMT Symbol Digit Modalities Score; HVLT-R Hopkins Verbal Learning Test Delayed Recall.

†: adjusted for age, sex, years of education, and CSF hemoglobin; Bold text: significant results
